# In-Depth Evaluation of Saliency Maps for Interpreting Convolutional Neural Network Decisions in the Diagnosis of Glaucoma Based on Fundus Imaging

**DOI:** 10.3390/s24010239

**Published:** 2023-12-31

**Authors:** Jose Sigut, Francisco Fumero, José Estévez, Silvia Alayón, Tinguaro Díaz-Alemán

**Affiliations:** 1 Department of Computer Science and Systems Engineering, Universidad de La Laguna, Camino San Francisco de Paula, 19, La Laguna, 38203 Santa Cruz de Tenerife, Spain; ffumerob@ull.edu.es (F.F.); iestevez@ull.edu.es (J.E.); salayon@ull.edu.es (S.A.); 2 Department of Ophthalmology, Hospital Universitario de Canarias, Carretera Ofra S/N, La Laguna, 38320 Santa Cruz de Tenerife, Spain; vtdac@hotmail.com

**Keywords:** saliency methods, glaucoma diagnosis, convolutional neural networks, deep learning, retinal fundus images

## Abstract

Glaucoma, a leading cause of blindness, damages the optic nerve, making early diagnosis challenging due to no initial symptoms. Fundus eye images taken with a non-mydriatic retinograph help diagnose glaucoma by revealing structural changes, including the optic disc and cup. This research aims to thoroughly analyze saliency maps in interpreting convolutional neural network decisions for diagnosing glaucoma from fundus images. These maps highlight the most influential image regions guiding the network’s decisions. Various network architectures were trained and tested on 739 optic nerve head images, with nine saliency methods used. Some other popular datasets were also used for further validation. The results reveal disparities among saliency maps, with some consensus between the folds corresponding to the same architecture. Concerning the significance of optic disc sectors, there is generally a lack of agreement with standard medical criteria. The background, nasal, and temporal sectors emerge as particularly influential for neural network decisions, showing a likelihood of being the most relevant ranging from 14.55% to 28.16% on average across all evaluated datasets. We can conclude that saliency maps are usually difficult to interpret and even the areas indicated as the most relevant can be very unintuitive. Therefore, its usefulness as an explanatory tool may be compromised, at least in problems such as the one addressed in this study, where the features defining the model prediction are generally not consistently reflected in relevant regions of the saliency maps, and they even cannot always be related to those used as medical standards.

## 1. Introduction

Explainability of deep learning systems is a research field of paramount importance in many areas where decisions must be carefully analyzed due to their complexity and/or implications. This is the case of medical image analysis, where the specialist needs to know the pieces of evidence that support the system’s decision so that they can be contrasted against her knowledge and experience. This paper presents a study of an important class of explainability techniques, called attribution/saliency maps, in the particular area of glaucoma diagnosis from fundus images. Therefore, the main question that this paper tries to answer is if the relevant image areas according to saliency maps computed on trained deep learning models for this particular problem (glaucoma) can be used by the specialists to support a diagnosis. A positive answer to this question is only possible if the evidence resulting from the attribution map can be aligned with the standard features that experts commonly analyze in medical images. This idea has led us to design an evaluation methodology that differs from other proposals, where saliency maps are scored case by case, comparing the signaled regions with the ones marked by experts (local interpretability).

In contrast, the proposed methodology evaluates the outcome of interpretability methods from a global perspective, rather than on a per-image basis. The aim is to estimate, for a trained model, a degree of coincidence between the image areas used in standard practice and the areas that are frequently relevant for the trained model. With this objective, we propose a new metric that we consider appropriate for this global interpretability analysis and in a context where there are standard relevant regions. The metric relies on the set of saliency maps obtained from a distribution of input images for the trained model, and in the standard relevant areas where the medical experts find shreds of evidence to support a glaucoma diagnosis.

Glaucoma is a disease that causes vision loss, as it leads to irreversible damage to the optic nerve. It is the second leading cause of blindness in the Western world, and is very difficult to diagnose, especially in its earliest stages of development, due to the absence of initial symptoms [1]. Thus, accurate systems with reliable interpretation methods for the automatic detection of glaucoma would mean a major positive change given the particular features of this illness and the importance of early identification.

**Figure 1:**
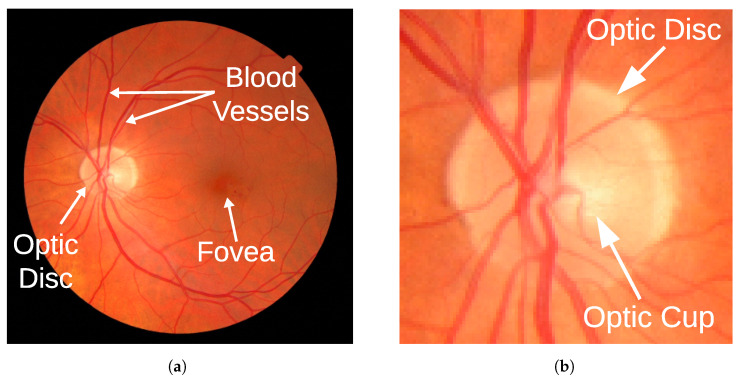
Sample color fundus image highlighting its main structures: optic disc, optic cup, fovea, and blood vessels. (**a**) Full fundus image with its main structures: optic disc, fovea, blood vessels; (**b**) A magnification of the image showing the optic disc and cup.

A photograph of the fundus of the eye is one of the most widely used tools in the diagnosis of glaucoma, since it is possible to appreciate the state of the structures of the eye as shown in Figure 1. The disease produces a structural change in the head of the optic nerve, an elliptical region called the optic disc that contains a central depression called the cup. Glaucoma causes progressive enlargement of the optic cup such that one method of estimating the disease with a fundus photograph is to measure the cup-to-disc diameter ratio (CDR) [2].

The visual assessment of these images is a very subjective operation and not always simple, especially in the most incipient cases of the disease. Therefore, automated methods can reduce costs and make quick and consistent predictions, helping the specialist in the diagnosis of this disease. Techniques based on deep learning (DL), such as convolutional neural networks (CNNs), and more recently, visual transformers, have proven to be very useful for solving computer vision problems [3]. In this work, we focus on CNNs, which have been one of the most researched DL models in recent years and have led to spectacular advances in their performance, surpassing classic machine learning techniques. Their main advantage is that they are capable of autonomously detecting and learning the most important features present in the image if their training is carried out with a large number of samples. Despite this success, these networks have a major drawback: a lack of transparency. CNNs are known to work very well, but it is not easy to understand why, since they do not provide an explanation for the decisions they make internally. They are often used as black boxes, even in medicine, where a human-friendly explanation of their behavior would be essential [4,5,6].

If we apply a CNN to perform diagnostic tasks on medical images, it would be very enriching to understand/visualize what high-level factors or features the network extracts from the images to make decisions. This knowledge could reinforce that of the medical specialist and even unveil new influential aspects in diagnosis hitherto unknown. Furthermore, if we want to fully integrate this type of system into clinical practice, we must achieve the confidence of the end user (the physician) in the decisions made by the network, and this can only be carried out by making the internal behavior of the system more transparent.

To try to explain this behavior, several visualization methods have emerged in recent years. The most popular, known as attribution or saliency methods, aim to identify those pixels of the input image that most influence the final prediction of the model. The result of the application of these methods is usually a heat map in which the pixels of the image that appear most relevant to the decision are highlighted [7].

There are several published works on the application of CNNs in the diagnosis of glaucoma that use these saliency maps. Most of them are limited to calculating the heat map corresponding to some example images to obtain an idea of which parts of them have been more determinant for the CNN used [8,9,10,11]. However, none of them perform an exhaustive analysis of these heat maps to know if they are reliable, coherent between different images or networks, coincident with the criteria of the medical specialist, or representative of the important areas of the image. The few publications that we have found focused on the detailed study of this type of technique are oriented to other medical problems, as will be discussed in Section 2.

Therefore, the present work aims to study, critically and in depth, the application of some of the most commonly used attribution methods in the interpretation of the behavior of CNNs trained to diagnose glaucoma utilizing eye fundus images. For this purpose, we performed a systematic application of these methods to determine the most relevant areas in the images and we analyzed different network architectures, as detailed in Section 3. As far as we are aware, we have identified a significant research gap, as there is currently no comprehensive publication addressing the specific subject of explainability in the context of glaucoma diagnosis using color fundus images, through the lens of saliency methods. Our primary contributions to the existing body of knowledge are as follows:

1. Assessment of saliency method suitability. We systematically assess the appropriateness and effectiveness of various widely recognized saliency methods in the context of glaucoma diagnosis using color fundus images. This involves a thorough examination of their applicability and performance metrics to determine the most suitable methods for this particular medical imaging problem.
2. Impact of CNN architecture and training data. We delve into a detailed analysis of the influence exerted by different convolutional neural network architectures and variations in training data on the resultant saliency maps. By systematically varying these factors, we aim to provide insights into the robustness and generalizability of saliency methods across different CNN configurations and datasets.
3. Correlation of saliency maps with medical criteria. Our research extends to an in-depth evaluation of the relevance of distinct regions within the images as identified by CNNs in making diagnostic decisions. Moreover, we establish correlations between these highlighted regions and established medical criteria for glaucoma diagnosis. This step is crucial for understanding the gap between algorithmic interpretations and clinically relevant features.

The rest of the article is structured as follows. Section 2 describes the related work. Section 3 refers to the materials and methodology used. Section 4 describes and discusses the experiments performed, and finally, Section 5 provides the conclusions.

## 2. Related Work

As already mentioned in the introduction, several research papers apply attribution methods to analyze the decisions made by CNNs for glaucoma diagnosis. However, these works lack an in-depth systematic analysis and evaluation of the heat maps obtained with these methods. In other medical problems, we have found some papers that address this issue from a more comprehensive and systematic point of view, although there are few.

Arun et al. [12] evaluate the (un)reliability of saliency maps for locating abnormalities in X-ray images for pneumothorax segmentation and pneumonia detection. They apply 8 saliency methods to the results of several CNNs. They conclude that the use of saliency maps in the high-risk domain of medical imaging warrants additional scrutiny. Similar conclusions were obtained in [13], where seven saliency methods were evaluated across multiple neural network architectures for chest X-ray interpretation. The popular GradCAM method performed better than the other saliency methods considered, but still, all seven provided significantly different explanations compared to the human benchmark. They also conclude that saliency methods have several important limitations.

In the field of ophthalmology, Singh et al. [14] present an interesting evaluation of 13 attribution methods. They train the InceptionV3 model to diagnose three retinal diseases (choroidal neovascularization, diabetic macular edema, and drusen) and calculate saliency maps with these attribution methods. The explanations obtained are evaluated by a panel of 14 clinicians to assess their clinical significance. The results showed that the most appropriate method for a specific medical diagnostic problem may be different from the one considered best for another problem. In a systematic investigation, Van Craenendonck et al. [15] conducted a comprehensive evaluation of saliency methods in the context of diagnosing diabetic retinopathy through fundus images. They trained several network models, including ResNet50, VGG16, and InceptionV3, and assessed the performance of 10 different saliency methods. Their experiments revealed significant disparities between the regions highlighted by heat maps and the annotations provided by expert clinicians. Ayhan et al. [16] used three different network architectures and trained CNNs to detect diabetic retinopathy and neovascular age-related macular degeneration from retinal fundus images and optical coherence tomography scans, respectively. They used various explanation methods and obtained a large set of saliency maps that were validated against clinicians. They found that the choice of CNN architecture and explanation method significantly influenced the quality of the saliency maps.

The research outlined in this paper aligns with the studies previously mentioned, yet it is specifically centered on the challenge of diagnosing glaucoma. In pursuit of this objective, we have examined nine distinct saliency methods and trained a total of twenty CNN models with four distinct architectures: VGG19, ResNet50, InceptionV3, and Xception.

## 3. Materials and Methods

### 3.1. Datasets

The dataset employed for training and evaluating various CNNs in this study comprises two components. Firstly, the publicly available image set RIM-ONE DL [17] is utilized, encompassing 172 images of glaucomatous eyes and 313 images of healthy eyes. Additionally, eye fundus images sourced from medical specialists within our research team, affiliated with the Hospital Universitario de Canarias, are included. This supplementary set consists of 191 glaucoma images and 63 images of healthy eyes. These images, which are not publicly available, were acquired with a Topcon TRC-NW8 multifunctional non-mydriatic retinograph. Patients with a diagnosis of primary or secondary open-angle glaucoma with untreated intraocular pressure greater than 21 mmHg were included in the study. The diagnosis of glaucoma was not only based on the observation of the eye fundus images but on the presence of reproducible defects in the white–white perimetry and/or morphological criteria based on a spectral domain optical coherence tomograph SD-OCT Spectralis with the glaucoma Premium and Posterior Pole module. Both eyes were included if they met the inclusion criteria. Subjects with concomitant ocular pathology other than glaucoma, lower visual acuity of 20/40, refractive error greater than five diopters of spherical equivalent or three diopters of astigmatism, level of false positives, negatives, and fixation errors equal to or greater than 25% in the visual field, were excluded from the study. Patients with hypoplastic or oblique optic nerves were also excluded. Considering the two sets, a total of 739 images have been used: 363 of glaucomatous eyes and 376 of healthy eyes. All these images have been annotated by two experts and include manual disc and cup segmentation.

For a more thorough validation of the CNNs, we have used other publicly available databases, namely REFUGE, DRISHTI-GS1, and G1020. The REFUGE challenge database [18] is composed of 1200 retinal images, of which 10% (120 samples) correspond to glaucomatous subjects, including primary open-angle glaucoma and normal tension glaucoma. The dataset also contains the ground-truth segmentation of the disc and cup. The DRISHTI-GS1 dataset [19] contains 101 fundus images with different resolutions and ground truth labels for the optic disc and cup. The G1020 dataset [20] is a large dataset of retinal fundus images for glaucoma classification. This dataset consists of 1020 high-resolution color fundus images and provides annotations of the ground truth for glaucoma diagnosis; optic disc and optic cup segmentation; vertical CDR; neuroretinal rim size in the inferior, superior, nasal, and temporal quadrants; and location of the optic disc bounding box. Importantly, the authors of the G1020 dataset acknowledge that this is a very difficult set to classify automatically because it represents fundus imaging in routine clinical practice and does not impose strict inclusion criteria on the images captured.

### 3.2. Deep Learning Models

In this section, we explain how the CNN models used in this work were trained. To do so, we describe how the training and test sets were obtained from the available data, the training strategy, and the parameters selected for each CNN. Finally, we show the performance attained.

In training the CNNs for this study, the initial set of 739 images underwent a random division into training and test sets, maintaining an 80/20 proportion, respectively. The training set comprised 290 retinographies of glaucoma and 301 of healthy eyes, while the test set included 73 retinographies of glaucoma and 75 of healthy eyes. Additionally, the training set was further partitioned into five distinct training and validation subsets, employing a 5-fold approach.

We selected four prominent convolutional neural network architectures, namely VGG19 [21], ResNet50 [22], InceptionV3 [23], and Xception [3], based on their widespread adoption and extensive utilization in the domain of glaucoma diagnosis and other medical fields. These architectures have also been evaluated in similar studies, such as the works analyzed in Section 2.

All specified neural network architectures are accessible in the Keras module of the Tensorflow v2 package [24]. To tailor these models to our specific problem, we modified the top layer of each network. The adaptation involved introducing a *DropOut* layer, succeeded by a *Flatten* layer, and then a *Dense* layer featuring 128 neurons with *ReLU* activation. Subsequently, another *DropOut* layer was added, followed by a final *Dense* layer with two outputs utilizing the *SoftMax* activation function.

For VGG19, the *DropOut* rate was set to 0.5, while for InceptionV3, ResNet50, and Xception, it was set to 0.2. Additionally, in these three networks, the *BatchNormalization* layers were maintained in inference mode to prevent the non-trainable weights from being updated during the training phase.

Concerning the size of the *Input* layer, it was configured as 224 × 224 × 3 for ResNet50 and VGG19, and 299 × 299 × 3 for InceptionV3 and Xception.

Our training strategy is the same for all the models. First, starting with the pre-trained weights from ImageNet, the base model was frozen and we trained the new top layer for 200 epochs using an *Adam* optimizer, with a learning rate of 1 × 10^−6^, and categorical cross-entropy as the loss function. Second, we unfroze the base model, except for the *BatchNormalization* layers, and trained the entire model end-to-end for 250 epochs, using the same optimizer, with a learning rate of 1 × 10^−5^, and the same loss function as before. In all cases, a batch size of 8 was used.

Regarding the pre-processing step, we implemented the pre-processing function included in Keras for each network. To mitigate over-fitting, we employed data augmentation on the input samples. This involved the introduction of random contrast adjustments (±0.3), random brightness variations (±0.3), horizontal random flipping, random rotation ($\pm 45^{\circ }$) with nearest fill mode, random translation (±0.05) both horizontally and vertically with nearest fill mode, and random zooming (±0.2) while preserving the aspect ratio, using nearest fill mode.

The final weights for each model were chosen from the epoch that maximized the validation accuracy on average among the five folds. This resulted in five different models per network architecture, totaling 20 models.

The trained CNN models were tested with the independent set we mentioned previously, which consists of 75 samples from healthy subjects and 73 from glaucoma subjects. The results achieved per network architecture and fold have been included in Table 1, highlighting those corresponding to the best performance per architecture in terms of balanced accuracy, which is the arithmetic mean of sensitivity and specificity [25]. Analogously, Table 2, Table 3 and Table 4 show the performance obtained by evaluating the trained models on the REFUGE, DRISHTI-GS1, and G1020 datasets. It is important to remark that these additional image sets have only been used for testing.

Although an exhaustive analysis of the performance of the trained CNN models is not the subject of this article, it can be seen that the models classify reasonably well the images of all the sets considered, except those of the G1020 dataset. The loss of performance achieved with this set is striking, confirming, therefore, what the authors of this dataset indicated in their publication, as commented in Section 2.

### 3.3. Saliency Methods

This paper evaluates a set of attribution/saliency methods for the problem of glaucoma diagnosis with CNNs trained with eye fundus images. These techniques aim to identify which features of the input image are most influential in the final prediction of the model. To do so, they generate attribution/saliency maps, which are maps in which the pixels of the image that seem most relevant to the decision are highlighted. In this study, we obtain the attribution from the model’s inferred class, whether it is correct or not. Therefore, in our interpretation of the attribution map, the most important thing is to find the features that are relevant to each particular model in the construction of its decision.

In [26] a very complete review of the currently existing techniques for interpreting the internal behavior of machine learning systems, including CNNs, is presented. In this work, two types of interpretability are differentiated, global interpretability and local interpretability. Global interpretability is attained when the user is able to understand how the model works at a global level by inspecting the internal structures and parameters of the model. On the other hand, local interpretability analyzes an individual prediction of the model and attempts to explain what input feature led to the particular decision corresponding to that prediction.

The attribution methods analyzed in this paper fall within the second category, as their objective is to provide an interpretation of the model’s decision for a particular sample within the domain, in our case a specific fundus image. However, the research strategy carried out also aims to achieve a global interpretation by studying the attribution results across a set of samples. This approach is intended to unveil insights into general aspects of the performance exhibited by the trained models. For this purpose, nine different attribution techniques, as outlined in Table 5, were examined. The selection of these saliency methods was driven by their widespread usage in analogous studies, as detailed in Section 2, where their effectiveness in elucidating model decisions and their contribution to the interpretability of deep learning models in medical image analysis was extensively evaluated.

The Grad, GBack, SGrad, SGrad2, and VGrad methods rely on the gradient calculation to obtain a sensitivity measure, i.e., how much a variation in the input contributes to a variation in the output. On the other hand, the IGrad, Occl, GCam, and SCam methods analyze how much of the CNN output can be attributed to the contribution of a feature. Therefore, the correct interpretation of the attribution maps must take this fact into account. In what follows, we briefly describe the basics of each of these methods.

The Gradient (Grad) method [7,27] is based on the calculation of the gradient, using the backpropagation algorithm together with automatic differencing to estimate the sensitivity of the model to each input. It is a fast method with a simple interpretation but has some important drawbacks. There is a strong dependence between the sensitivity recorded for one feature and the value of the other feature due to the nonlinearity of the models, which makes it a very noisy method. Moreover, when the model is saturated for a subset of its features, the gradient remains zero even for large variations of the inputs [28].

The Guided Backpropagation (GBack) method [29] is another gradient-based visualization technique that allows only the propagation of positive gradients through the *ReLU* activation function, changing the negative gradient values to zero. The elimination of these negative values reduces the noisy appearance of the attribution map.

The SmoothGrad (SGrad) method [30] attempts to reduce the noise of the saliency maps through the perturbation of the original image by adding noise to this image before calculating the gradient. This is repeated N times and the resulting gradients are averaged, leading to a reduction of the apparent noise in the attribution map while still emphasizing important regions. Variants of SmoothGrad are SmoothGrad Squared (SGrad2), in which the gradient is squared before averaging, and VarGrad (VGrad) [31], where the attribution value is obtained from the variance of the gradients.

The Integrated Gradient method (IGrad) [32] integrates the gradient attribution map between a baseline image and the input image, and the result is multiplied by the difference between the input image and the baseline. The baseline image can be a black image, an all-white image, or a random image. It is similar to the SmoothGrad method because it works from a set of perturbed images. In the SmoothGrad method, they are perturbed by adding noise, while in the IGrad method, this perturbation is a linear interpolation between the baseline and the original image.

The Occlusion method (Occl) [33] attempts to discover which features of an input image are the most influential in the network decision. They start from the assumption that the contribution of a feature can be determined by measuring how the prediction changes when that feature is occluded. The key question is as follows: Which parts of the input image, if the model cannot see them, would change the final prediction the most? Its simplest implementation is to replace a region of the input image with a uniform square of a given color, but noise or specific textures can also be used as replacements. The region is slid over the entire image, and the differences in prediction values for the reference class are given as the estimated saliencies.

Both occlusion-based and gradient backpropagation-based interpretation methods do not consider explicitly the intermediate layers of the network, which may contain important information about its interpretability. There is a third type of method that investigates what happens in the hidden layers of the network, to try to determine which features of the input are the most relevant. This is the case of GradCAM (GCam) [34] and ScoreCAM (SCam) [35], which generate saliency maps by combining the feature maps of intermediate layers. GCam uses the gradient information flowing into the last convolutional layer to assign importance values to each neuron, for a given output decision. These importance values or weights are applied to compute the weighted sum of the feature maps generated by each neuron of this last convolutional layer, thus obtaining the saliency map. SCam applies an occlusion technique on the input image using the feature activation maps of the last convolutional layer of the network as masks (previously adjusting the size of these feature maps to the size of the original image). In this way, a score is obtained for each feature map, which indicates the importance of that feature map in the final predicted class. As in GCam, these scores are used as weights to calculate the weighted sum of the feature maps and thus obtain the final saliency map. The authors of SCam claim that this method is better than GCam because, by not relying on gradients, it avoids irrelevant noise and generates cleaner and more meaningful explanations.

**Figure 2:**
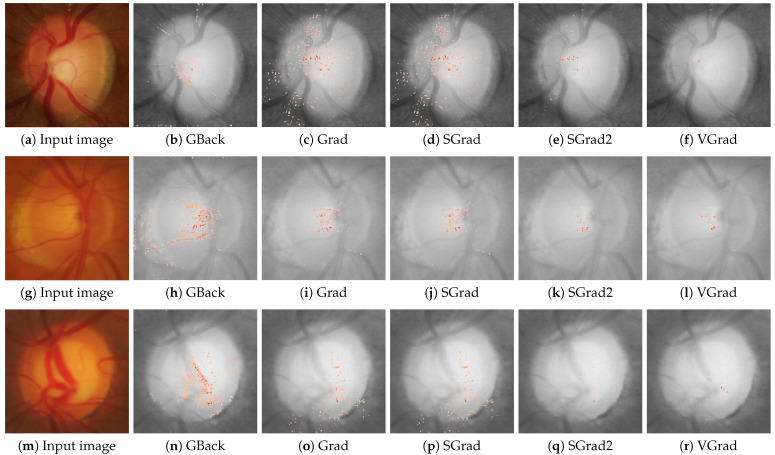
Saliency maps from GBack, Grad, SGrad, SGrad2, and VGrad are displayed for three cases: a scenario confidently classified as healthy with a probability of 1.0 in the first row, a highly uncertain case with a probability of 0.61 for being healthy in the second row, and a case confidently classified as glaucoma with a probability of 1.0 in the third row. The first column shows the input image for the respective case.

**Figure 3:**
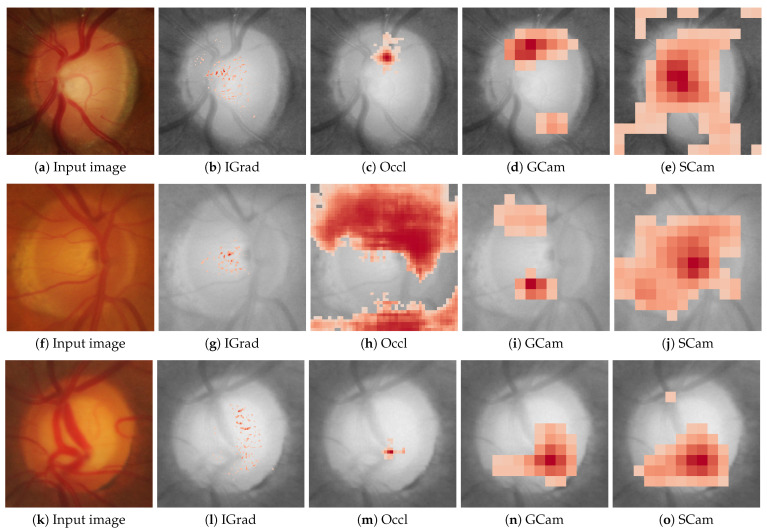
Saliency maps from IGrad, Occl, GCam, and SCam are displayed for three cases: a scenario confidently classified as healthy with a probability of 1.0 in the first row, a highly uncertain case with a probability of 0.61 for being healthy in the second row, and a case confidently classified as glaucoma with a probability of 1.0 in the third row. The first column shows the input image for the respective case.

Figure 2 and Figure 3 show examples of the saliency maps obtained with the different attribution methods considered. Figure 2 groups the methods based on gradient backpropagation, while Figure 3 shows the saliency maps of the remaining methods. The results of the CAM methods are shown at the resolution at which they were actually calculated instead of interpolating them, as is usually carried out, to avoid introducing distortions in the results.

### 3.4. Evaluation Methodology

In order to discover the most relevant information contained in the saliency maps, the evaluation methodology that we have followed in this work is partially inspired by the one used in [12]. In that study, the authors calculate for each saliency map what they call the “localization utility”, which is a measure of the coincidence between the areas of the original image marked as relevant in the saliency maps and those studied by the medical specialists in their diagnosis. Therefore, for a saliency method to be considered useful, the maximum values of the saliency map must be located in the image area indicated by the experts in the ground truth.

The study performed in [12] was carried out with radiology datasets, in which the image areas of diagnostic interest are well determined and localized. However, for specialists diagnosing glaucoma, it is not so easy to precisely specify the most relevant regions in retinal images due to the lack of a reliable diagnostic biomarker. The diagnosis of glaucoma is usually based on the joint analysis of the patient’s clinical history and the results of various structural and functional tests. This is the main reason why, in this work, we have followed a different approach for the evaluation of the localization utility of the CNNs used.

**Figure 4:**
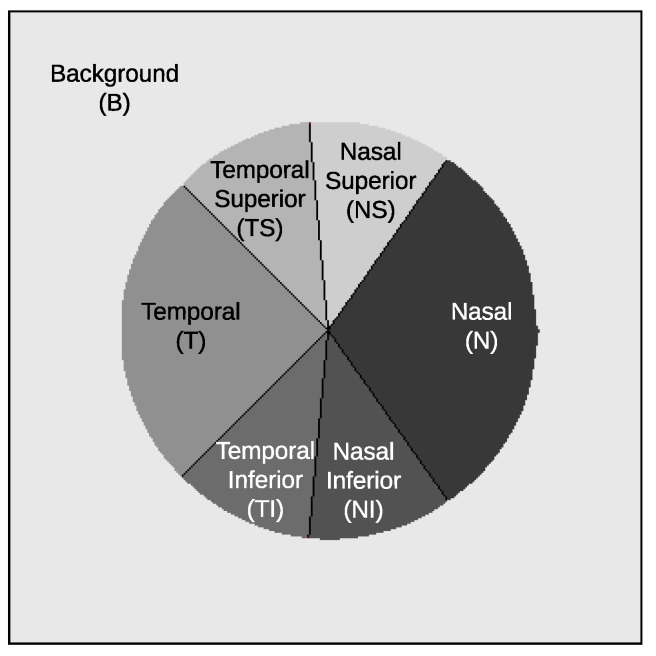
Illustration depicting the partitioning of the optic disc into six sectors: nasal superior (NS), nasal (N), nasal inferior (NI), temporal inferior (TI), temporal (T), and temporal superior (TS). An additional sector is dedicated to the background (B). This visual representation pertains to the right eye. Note that for a left eye, the sectors would be horizontally mirrored.

As mentioned in the introduction, glaucoma is characterized by a progressive enlargement of the optic cup area, leading to a narrowing of the neuroretinal rim [2]. In glaucoma, the enlargement of the optic cup occurs in all directions, but generally, some areas are affected earlier than others. Thus, in eyes with modest glaucomatous damage, rim loss is found mostly at the temporal inferior and temporal superior regions. In eyes with moderately advanced glaucomatous atrophy, the temporal region is the location with the most relative marked rim loss. In cases of highly advanced glaucoma, the least pronounced rim loss is typically observed in the nasal disc area, while the nasal inferior region tends to exhibit greater impairment than the nasal superior region. Examples of a healthy case and an advanced glaucomatous case can be seen in Figure 2a,m, respectively. In order to identify these zones of the image in a standard way, some sectors are defined in the optic disc [36], as shown in Figure 4. This sectoral identification allows us to relate the most relevant zones of the saliency maps with the areas of interest for medical specialists, which facilitates the calculation of the localization utility of the analyzed method. In addition, the sector analysis allows us to dispense with the pixel level and perform a higher-level study that is less noisy and of greater meaning for the specialist. In [15], a discretization of the problem is also proposed but through an arbitrary regular grid.

Formally, given an input image *I* and a model *f*, a saliency or attribution method, denoted as $S=S_{I}^{f}\left(p\right)$, can be defined as a function that assigns a relevance score to each pixel *p* in the image. Consequently, we transition from this conventional saliency map to a discretized counterpart, denoted as $Sd=Sd_{I}^{f}\left(sec\right)$, which allocates relevance scores to sectors associated with the image *I*. This is computed as follows:

(1) $$\begin{array}{c} Sd_{I}^{f}\left(sec\right)=\frac{|M_{sec}\cap M_{sal}|}{|M_{sal}|},sec\in \left\{N,NI,TI,T,TS,NS,B\right\} \end{array}$$

Here, $M_{sec}$ represents the binary mask for pixels encompassed within the sector being examined, and $M_{sal}$ is the binary mask derived by applying a threshold to the saliency map. This threshold retains pixels with values surpassing 75% of the maximum value, specifically:

(2) $$\begin{array}{cc} M_{\mathrm{sal}}\left(p\right) & =\left\{\begin{array}{ccc} 1 & \mathrm{if} & S(p)\geq 0.75max(S(p))\\ 0 & \; & \mathrm{otherwise} \end{array}\right. \end{array}$$

**Figure 5:**
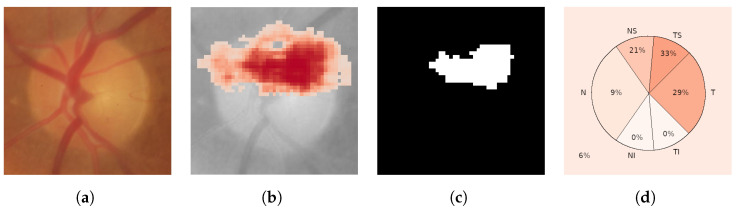
Example demonstrating the conversion of a saliency map into a discretized saliency map for the respective sectors. In this specific instance, the majority of the information significant for the network is located in the temporal superior, temporal, and nasal superior sectors. (**a**) Input image; (**b**) saliency map; (**c**) saliency mask; (**d**) discretized saliency map.

Therefore, the saliency score of each sector is the fraction of mask pixels that fall within that sector. The choice of the threshold (0.75 of the maximum value of the saliency map) was made empirically. Neither the maximum value of the saliency map nor the average value per sector has been used as a threshold because we have found that these choices lead to noisy and unreliable results. In Figure 5, we exemplify the transformation of a saliency map into a discretized saliency map for the respective sectors. This conversion process is performed following Equations (1) and (2).

In addition to discovering the most relevant sectors from the information contained in the saliency maps, we have carried out a complete comparative study between saliency maps to measure the degree of agreement between saliency methods for a specific model, the degree of agreement between saliency methods for models with the same architecture, and the degree of agreement between saliency methods for models with different architectures.

Spearman’s rank correlation [37] served as the method for quantifying the level of agreement among the saliency maps derived from all the experiments conducted within this study. Furthermore, to have a baseline of what can be considered a reasonable correlation according to [38], we performed two additional tests for each of the 20 trained models: a model parameter randomization test and a label randomization test.

The model parameter randomization test compares the output of a saliency method on a trained model with the output of the same saliency method on an untrained network of the same architecture, with its parameters randomly initialized. In [38], it is pointed out that if the saliency method is truly useful and representative, it must depend on the learned parameters of the model, and therefore, it is expected that its output differs substantially between the two cases. A high correlation between the outputs may indicate that the saliency map remains unresponsive to the characteristics of the model, which is an undesirable outcome. This basic requirement can be used to rule out saliency methods.

In the label randomization test, the class labels are randomized. For this purpose, the models are retrained by changing the labels of 50% of the samples with which they are trained. According to [38], a high correlation between the saliency maps of well- and poorly trained models is also undesirable, and if it occurs, it may be an indication to discard that saliency method.

## 4. Experimental Results and Discussion

Different experiments have been carried out with the 20 CNN models trained with our dataset (see Section 3.2 for more information), from the four architectures previously described and the nine selected attribution methods. Details of these experiments can be found in the following subsections. First, we present the experiments that verify the validity/representativeness of the selected attribution methods, and then, we thoroughly analyze the results obtained with them. All the results reported in this section were achieved by testing the models on our dataset, except for Section 4.6, where we additionally test them using the third-party datasets described in Section 3.1.

### 4.1. Model Parameter Randomization

**Figure 6:**
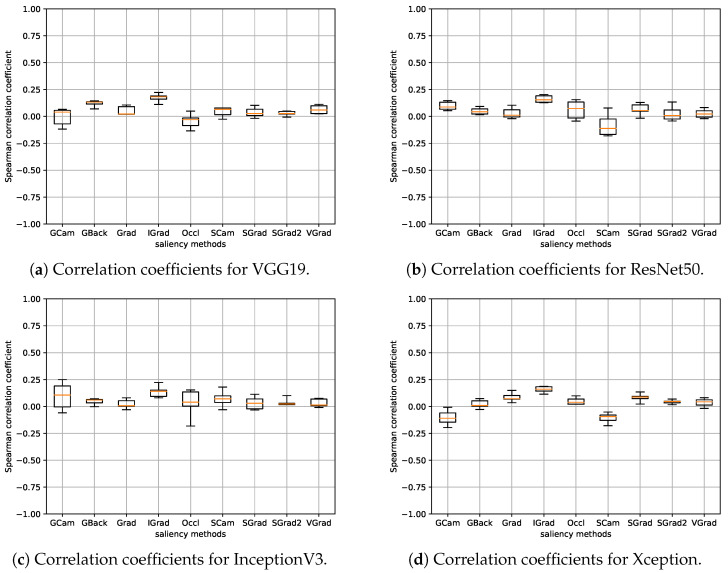
Boxplots depicting the correlation coefficients between discrete saliency maps of the randomized models and their original counterparts. A distinct boxplot is presented for each saliency method and architecture. Each boxplot encapsulates the five correlation values stemming from each fold per architecture.

In this section, the experiments of the model parameter randomization test are presented. As already indicated in Section 3.4, for the calculation of the correlation, the values of the saliency maps at the pixel level have not been used directly, but the values are calculated by sectors using Equations (1) and (2). This is the same for all experiments performed in this work. Therefore, since we have seven saliency values in each image (with the background considered as one additional sector) and 148 test images, the Spearman rank correlation for each pair of models is calculated, effectively, between two vectors of 1036 elements each. Figure 6 shows the boxplots for all the saliency methods. Each boxplot has been computed from the five correlation values obtained by considering the model corresponding to each fold per architecture. The results for the different architectures are shown separately.

**Figure 7:**
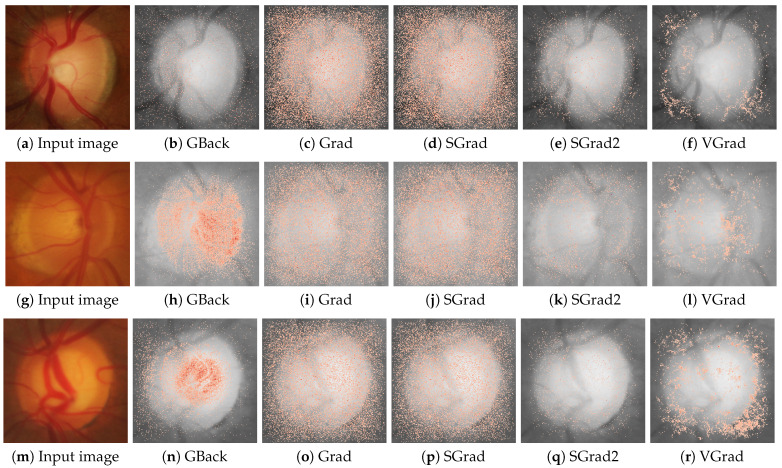
Saliency maps from GBack, Grad, SGrad, SGrad2, and VGrad obtained for the models with randomized weights in the same three cases as in Figure 2. The first column shows the input image for the respective case.

**Figure 8:**
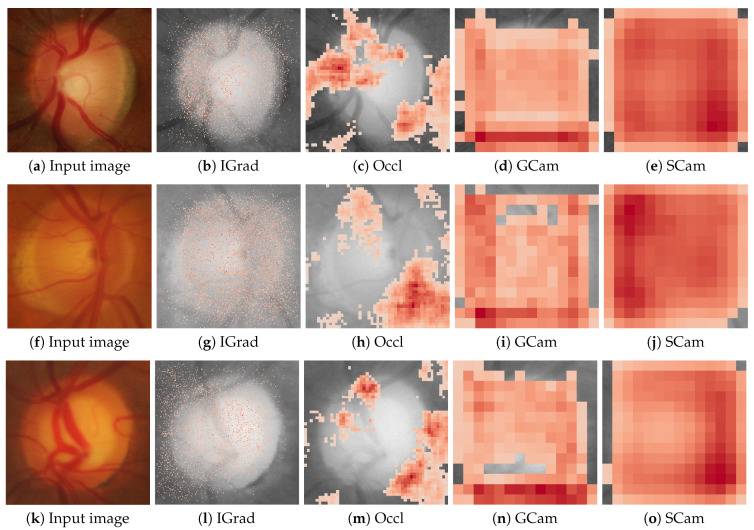
Saliency maps from IGrad, Occl, GCam, and SCam obtained for the models with randomized weights in the same three cases as in Figure 3. The first column shows the input image for the respective case.

It can be seen that, in general, the correlations between the methods and their randomized equivalents are low, with a maximum value that, at most, is around 0.25 in absolute value. Hence, we consider that the basic requirement of dependence on the learned parameters of the model seems to be fulfilled in all cases, and therefore, it is not necessary to discard any of the methods. Additionally, Figure 7 and Figure 8 display the saliency maps obtained with the different methods for the randomized model in the same three cases as Figure 2 and Figure 3. The differences are quite noticeable, with the saliency maps highlighting pixels that, in general, are scattered over the entire image.

### 4.2. Data Randomization

In this section, the experiments of the class label randomization test are presented. Let us recall that in this test what is randomized are the class labels. Once again, a high correlation between the saliency maps of well-trained models (with the original data) and wrongly trained models (with random class labels) is also undesirable. If it occurs, it can be considered a reason to discard the corresponding saliency method.

**Figure 9:**
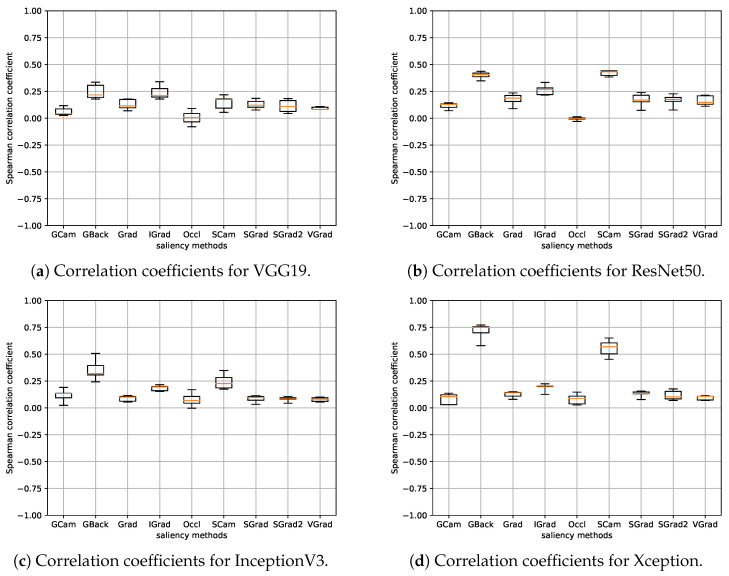
Boxplots depicting the correlation coefficients between discrete saliency maps of the models trained with randomized labels and their original counterparts. A distinct boxplot is presented for each saliency method and architecture. Each boxplot encapsulates the 5 correlation values obtained from each fold per architecture.

In these experiments, we also considered the seven saliency values in each of the 148 test images, calculating the Spearman rank correlation for each pair of models (the original model and the model retrained with the mislabeled samples). Figure 9 displays the boxplots for all the saliency methods. Each boxplot has been generated from the five correlation values obtained by considering the model corresponding to each fold per architecture. The results for the different architectures are presented separately.

In this case, we can observe that some methods present higher correlations than in the previous experiment. In particular, GBack and SCam achieved median correlation values close to or above 0.50 for ResNet50 and Xception, even reaching 0.75 in the case of GBack. Consequently, we have decided to discard these two methods in subsequent experiments. It is important to note that the GBack method also did not pass the sanity checks carried out in [38].

### 4.3. Correlation between Saliency Methods for a Specific Model

**Figure 10:**
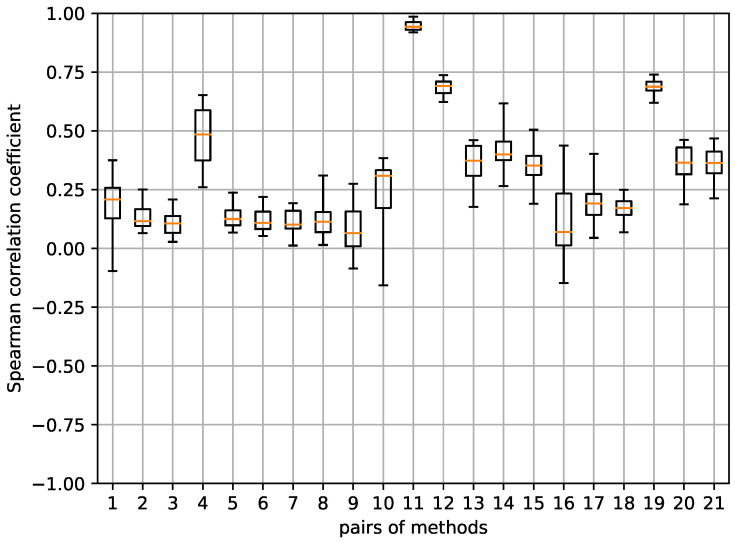
Boxplots illustrating the correlation coefficients for every pair of saliency methods.

We calculated the correlation between the saliency maps produced by the 20 models in our study. Figure 10 displays the resulting correlation coefficients, with each boxplot summarizing the statistical information for this analysis. These distinct boxplots were derived from the 20 correlation values calculated for pairs of saliency methods, encompassing all possible pair combinations listed in Table 5 and arranged alphabetically, excluding GBack and SCam, as previously noted. In this section, we did not differentiate between the various network architectures since the obtained results were similar. Additionally, we have included Table 6, which details some of the selected pairs of methods, sorted in descending order of their median correlation values, including those with the highest correlations.

It can be seen that saliency methods of the same type record the highest correlation values. Gradient backpropagation-based methods are the ones that appear in almost the entire table, as they exhibit the highest correlation. In contrast, GradCAM (GCam) and Occlusion (Occl) methods do not appear in the table because their correlation with the others and with each other is low. This approximately aligns with what was observed in the examples in Figure 2 and Figure 3.

It should be noted that for distinguishing between high and low correlation values, we can use as a reference baseline the ones obtained in the case of random parameter models with values in the range of 0.25, as previously seen.

The results found can be considered, to a certain extent, logical, since the methods related by their very nature are the ones that show a greater correlation. However, it begs the question of whether an even higher correlation could be expected, in general, since the saliency maps are computed on the same model trained with the same images.

Since there is no such thing as a ground truth of the saliency maps to be obtained, we cannot rely on any objective basis to consider one result better than another. However, we believe that the low correlation found in most cases may be because, depending on what is specifically computed in each type of method, different parts of the image may be highlighted without necessarily being mutually exclusive results. For example, the problem formulation is different in the case of a gradient-based backpropagation method compared to an occlusion-based method, as discussed in Section 3.3. Another issue is whether what is highlighted may have a higher or lower correlation with what may be of interest in the context of the considered pathology. This point will be addressed in Section 4.6.

### 4.4. Correlation between Saliency Methods for Models with the Same Architecture

**Figure 11:**
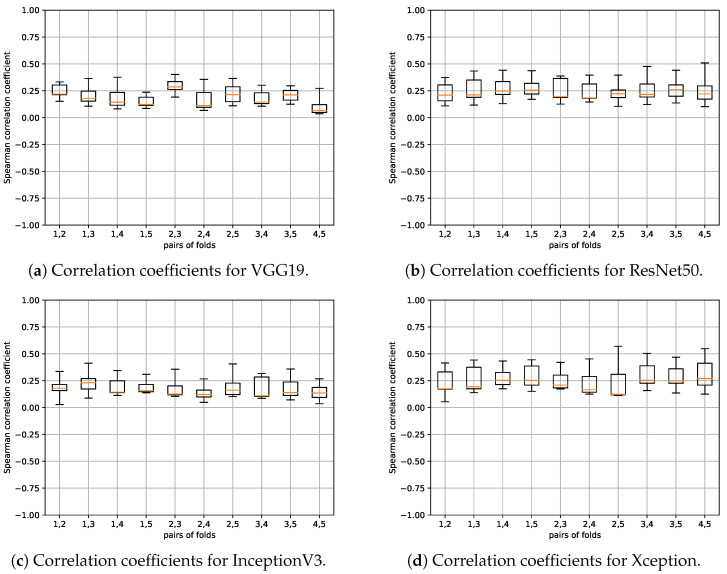
Boxplots representing the correlation coefficients between the different pairs of folds of each architecture for the seven saliency methods under investigation.

We computed the correlation between saliency maps across the five folds under examination. Figure 11 illustrates the outcomes, with each boxplot representing the correlation values among the seven saliency methods for pairs of folds within the same network architecture.

The response of the saliency methods to model changes in VGG19 and InceptionV3 is similar, with median values between 0 and 0.25, in general, and maximum values bordering 0.5 in some pairs. For both ResNet50 and Xception, the medians were higher, with maximum values above 0.5 in some cases. Xception appears to be the least sensitive to model change when these methods are applied to it.

We have identified a single comparable study in this context, conducted by Arun et al. in 2020 [12], which focused on employing X-ray imaging for diagnosing pneumonia. In their research, they undertook an evaluation of the repeatability of two distinct instances of the InceptionV3 architecture. These instances were randomly initialized and trained until convergence. Their published findings revealed significant variations across various saliency methods. Methods that yielded higher correlation values between the saliency maps of the two models were considered more favorable.

In our study, we also observed elevated values in the upper range of the box-and-whisker plots for certain saliency methods. However, we caution against interpreting these values as a direct measure of method superiority. It is important to recognize that these high values may arise due to the presence of image areas containing redundant information that can equally distinguish between the two classes. Consequently, neural networks trained under such circumstances may exhibit dissimilar training trajectories yet still yield similar performance. In such cases, a saliency method can appropriately highlight this scenario, indicating a discrepancy resulting from differing training processes rather than a flaw in the method itself.

### 4.5. Correlation between Saliency Methods for Models with Different Architectures

**Figure 12:**
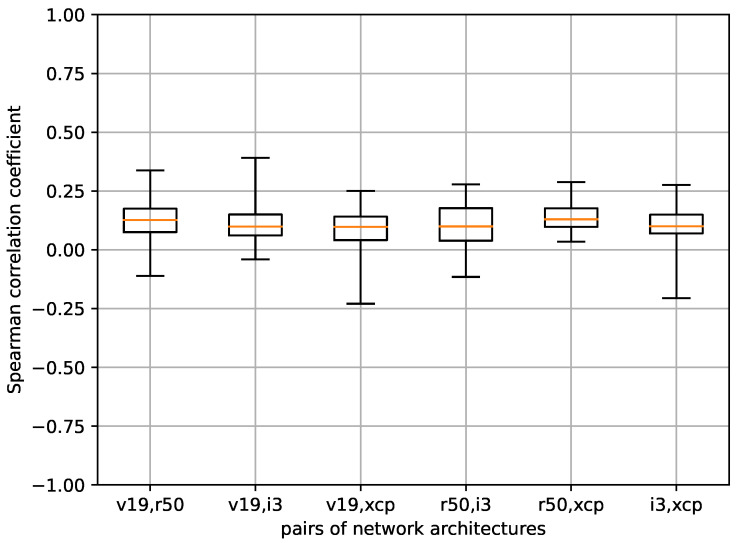
Boxplots illustrating the correlation coefficients for each pair of network architectures.

We computed the correlation among saliency maps for the four network architectures under scrutiny. Figure 12 visually presents the correlation coefficients, with each boxplot derived from the seven saliency methods for pairs of architectures within the same fold. In this experiment, the objective is to assess the level of concordance between saliency maps generated by models trained on distinct architectures using the same data.

Negative correlations can be found in all architecture pairs except ResNet50 and Xception. In general, the maximum correlation values achieved are lower compared to the previous scenario. In all cases, the average correlation values are around 0.125. In general, the results depicted in Figure 12 align with reasonable expectations, as lower correlations were anticipated when changing architectures, compared to the assumptions explored in the previous section. It is evident that altering the architecture has a more pronounced impact on correlation levels than changing the fold.

Once again, a closely related study was conducted by Arun et al. [12]. Their research also demonstrates that correlations between models of the same architecture (repeatability) tend to be higher than correlations between models of different architectures (reproducibility) across all considered saliency methods.

### 4.6. Relevance of Sectors in Neural Network Decisions

As previously elaborated in Section 3.4, a primary objective of this study is to determine the image regions that exert the greatest influence on the outcomes of the trained neural network models. To accomplish this, we have employed the regions within the optical disc that clinicians regard as standard, a consistent approach utilized throughout our experiments, as discussed in previous sections.

Section 4.3, Section 4.4 and Section 4.5 have delved into calculating correlations between saliency maps under various assumptions. In this section, we introduce a global relevance metric for each sector. This metric is calculated by determining the likelihood, within a specified test dataset, that the highest relevance value generated by a saliency technique for a particular image falls within the designated sector. The relevance value itself is derived using Equation (1). Notably, for this probability calculation, we differentiate between the neural network architectures, while considering all folds collectively.

**Figure A1:**
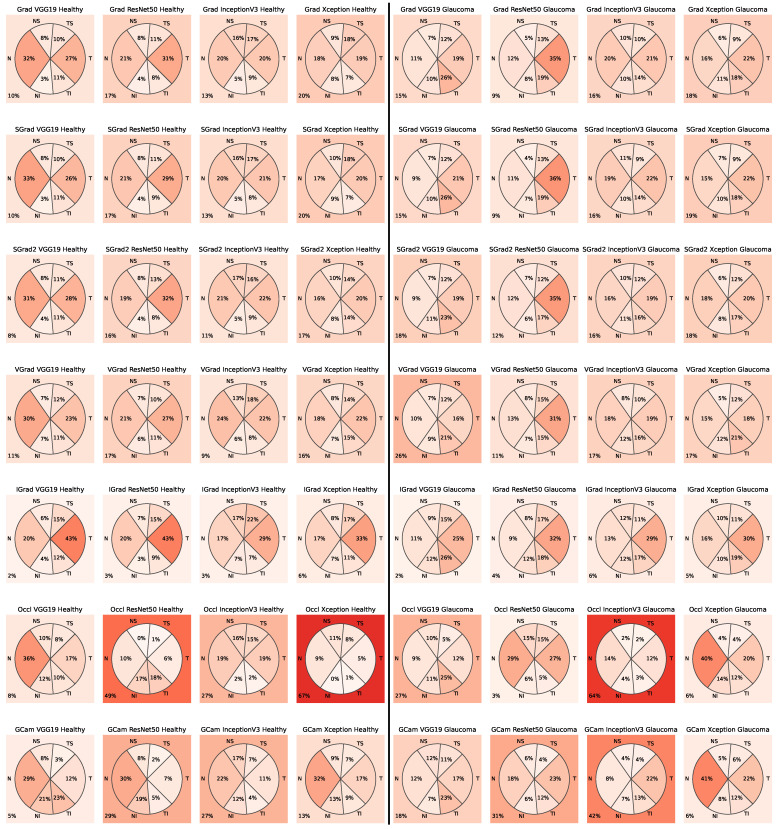
Global relevance values by sectors obtained with our dataset for the different network architectures and attribution methods, divided into healthy and glaucoma as classified by the networks. The color scheme visually represents relevance values, with lighter red tones indicating lower values and darker tones signifying higher values.

**Figure A2:**
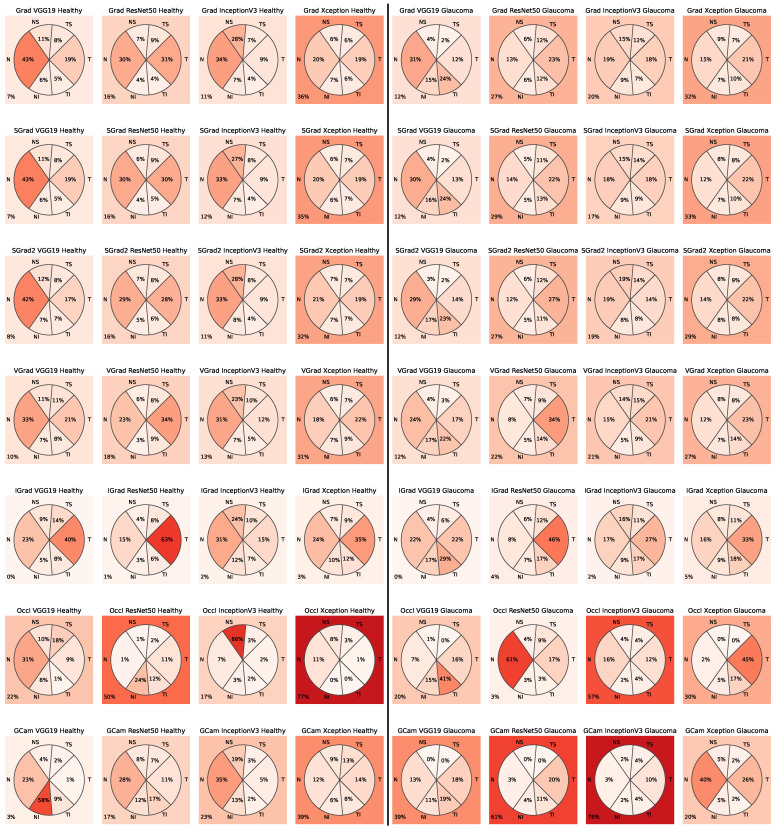
Global relevance values by sectors obtained with the REFUGE dataset for the different network architectures and attribution methods, divided into healthy and glaucoma as classified by the networks. The color scheme visually represents relevance values, with lighter red tones indicating lower values and darker tones signifying higher values.

**Figure A3:**
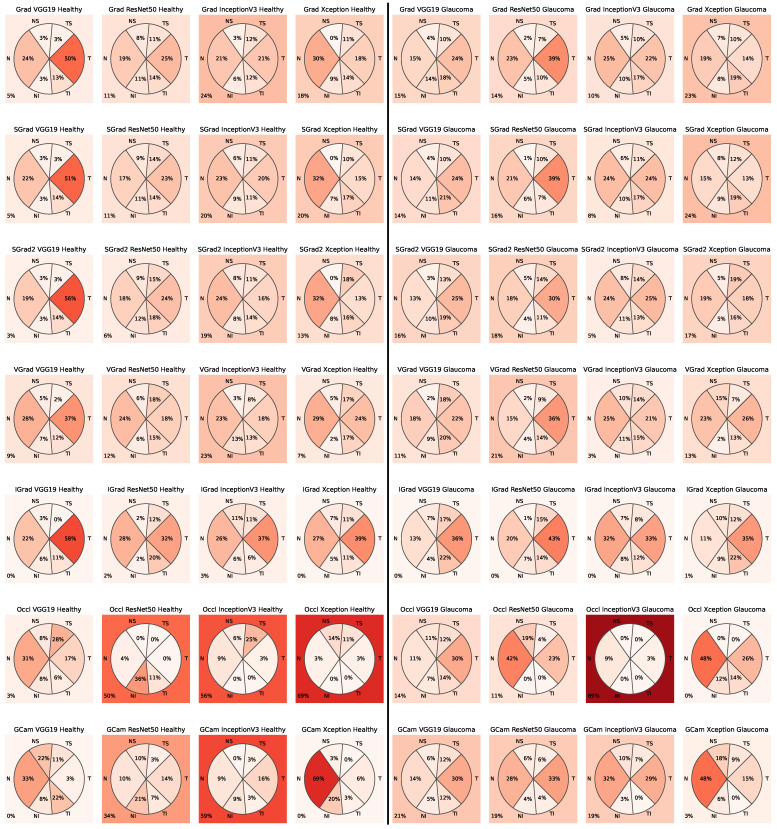
Global relevance values by sectors obtained with the DRISHTI-GS1 dataset for the different network architectures and attribution methods, divided into healthy and glaucoma as classified by the networks. The color scheme visually represents relevance values, with lighter red tones indicating lower values and darker tones signifying higher values.

**Figure A4:**
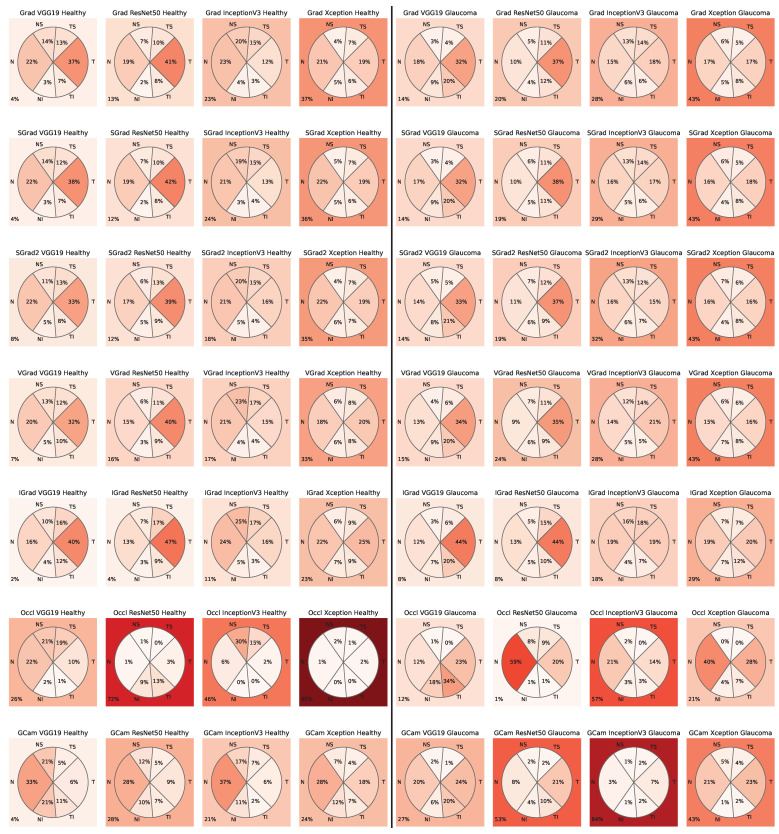
Global relevance values by sectors obtained with the G1020 dataset for the different network architectures and attribution methods, divided into healthy and glaucoma as classified by the networks. The color scheme visually represents relevance values, with lighter red tones indicating lower values and darker tones signifying higher values.

Figure A1, Figure A2, Figure A3 and Figure A4 in Appendix A show the global relevance values by sectors for each of the test datasets evaluated, and for the different network architectures and attribution methods, separating the data between cases classified by the network as glaucoma and those predicted as healthy. It can be seen that there exist differences depending on the saliency method and architecture used, although some similar patterns can be seen among the methods that rely more directly on gradient backpropagation: Grad, SGrad, SGrad2, and VGrad. The IGrad method also produces some similarities with the previous ones in terms of the sectors it considers most relevant, although it tends to highlight the background sector less in all cases. This is in agreement with the results we saw in the previous sections, especially those of the correlation between methods for a specific model in Section 4.3.

In addition, as mentioned in Section 3.1, we performed a validation of the models with other external sets: REFUGE, DRISHTI-GS1, and G1020. For the latter set, G1020, we removed from the study those images in which the optic nerve could not be fully seen, in order to correctly perform the calculation of the global relevance per sector. In total, 27 images of normal subjects and 13 of glaucoma were removed, leaving us with 697 normal and 283 glaucoma images. It should also be noted that for the calculation of the attribution maps on these sets, we only used the fold that obtained the best balanced accuracy according to the results presented in Section 3.2, to reduce the computation time given the volume of images they contain.

Besides the figures provided in Appendix A, for a comprehensive and coherent analysis, we have computed the average values presented in Table 7. This table consolidates data from all methods and architectures, offering a holistic perspective.

From the observation of these data, we can highlight the following:

1. The background, nasal, and temporal sectors are, in general, the most relevant for all sets and classes, but the importance of each of the sectors varies depending on the specific case.
2. The temporal inferior sector has approximately twice the probability of glaucoma than normal in our dataset and REFUGE, unlike the rest of the datasets, whose probability remains closer between classes. In our dataset, this sector has the highest probability of glaucoma cases compared to the rest of the sets. Perhaps this may be giving a clue to the performance difference between our dataset and the rest.
3. A common characteristic of all datasets seems to be the low probability, in general, of the nasal inferior sector. On the other hand, the nasal superior and temporal superior sectors also generally present a low probability, although higher than the nasal inferior.
4. In our dataset, the most globally relevant sector was the temporal sector, being the most predominant in both classes. Moreover, the temporal inferior sector is the second most important in glaucomas. This does not occur in any other dataset. The temporal and temporal inferior sectors, being the most globally important for glaucomas, approximately align with the medical criteria, except for the temporal superior region, whose relevance falls behind the background and nasal sectors.
5. Additionally, for our dataset, a Spearman correlation coefficient of 0.43603 (*p*-value < 0.001) was obtained between the overall relevance by sectors of samples classified as healthy and those classified as glaucoma. This indicates a moderate, or moderate to low, correlation between them.
6. In REFUGE, the most relevant sector was the nasal, but its relative importance varies by class. It is the most likely in normals but the third most likely in glaucomas.
7. In DRISHTI-GS1, the most important sector was the temporal sector, which maintained its position in both classes. The rest of the sectors in DRISHTI-GS1 also maintain their position unchanged by class.
8. In G1020, the most relevant sector was the background. It does not change its relative position by class and remains in first position in both, despite being more likely in glaucomas than in normal cases.
9. The behavior observed on average for DRISHTI-GS1 and G1020 could derive from a difference in the distribution of the data, which may have caused the loss of performance that our models produced when evaluated on these samples.

From what was highlighted above, it may be surprising that the background sector is one of the most relevant. In this regard, it is worth mentioning the study carried out in [39], where a series of experiments with this type of images are performed, hiding increasingly larger parts of the retina centered on the optic disc, until it is completely hidden. They conclude that there may be significant information outside the optic disc, such as the retinal nerve fiber layers, which may allow this type of model to detect glaucoma or even to estimate the vertical cup-to-disc ratio. In our case, something similar could be occurring with the trained models, which may have relied on information present in the surroundings of the disc to perform the prediction. Moreover, the experiments performed in [40] show that convolutional neural networks trained with ImageNet are strongly biased towards texture recognition rather than shape recognition. Note that, as explained in Section 3.2, our models have been trained starting from the ImageNet pre-trained weights, as usual when a large number of samples is not available, to avoid the over-fitting phenomena and to obtain better performance. So, another possible explanation is that some kind of calculation is being performed based on the texture of these images, where the background could play a relevant role.

## 5. Conclusions

The main conclusion we draw from the analysis carried out is that saliency maps are difficult to interpret and often provide rather unintuitive results in this particular problem. Regarding the degree of coincidence in the evaluation of the importance of the sectors of the optic disc, it seems that there is not much agreement between standard medical criteria and the decisions of neural networks, except in the particular case of our dataset for the glaucoma class. In this particular case, the lower and upper sectors of the disc are the ones that generally stand out in most saliency maps and architectures, which is in agreement with medical criteria. However, this consistency is lost when the trained models are confronted with other data sources. Here, the accuracy of the models is decreased and, even with a slight reduction, the previously described alignment with the medical standards disappears. This leads us to think that there is a relationship between the performance derived from the generalization capacity of the model and the consistency of the relevant areas in the saliency maps. Consequently, it may also influence the agreement with established medical criteria.

However, other factors could produce disagreement with the expected explanations. On one hand, saliency map techniques have their caveats, for example, it is well known that in the case of gradient-based techniques, saturation of the model can easily diminish the importance assigned to a relevant area. On the other hand, the model could be learning novel features or interactions between already known ones. For this reason, we believe that the results obtained in this paper should not be seen as a disadvantage of the application of deep learning systems for glaucoma diagnosis from fundus images, but on the contrary, as evidence of the absence of clear biomarkers in the images. This leaves the problem very open to be explored in different ways by humans and machines, unlike what occurs with other types of pathologies.

Finally, we need to address the practical implications of these results. In short, the use of saliency maps as tools for explainability in a problem like this should be handled very carefully to avoid incorrect interpretations. Deep learning models can be accurate predictors of glaucoma in fundus images, but a direct human comprehensible explanation that summarizes all the factors that the model is considering is not always possible. From an intuitive point of view, the model is probably perceiving the image globally as we perceive textures: there is no specific collection of spatially well-defined and consistently located features that distinctly differentiate one texture from another. Alternative approaches seek a more balanced integration of deep learning models’ predictive accuracy and interpretability. One such example is the use of “surrogate models,” wherein a more easily explainable model, like a high-level decision tree based on standard geometric features, is trained to mimic the predictions of a pre-trained deep learning system using a set of images [41].

## Figures and Tables

**Table 1:** Results obtained by training and testing with our dataset, according to different metrics. Results corresponding to the maximum balanced accuracy (B. Accuracy) are highlighted in bold.

Network	Fold	Sensitivity	Specificity	Accuracy	B. Accuracy	F1 Score
VGG19	1	0.8767	0.9467	0.9122	0.9117	0.9078
VGG19	2	0.9726	0.9467	0.9595	0.9596	0.9595
VGG19	3	0.9041	0.9733	0.9392	0.9387	0.9362
**VGG19**	**4**	**0.9863**	**0.9467**	**0.9662**	**0.9665**	**0.9664**
VGG19	5	1.0000	0.9200	0.9595	0.9600	0.9605
ResNet50	1	0.9315	0.9067	0.9189	0.9191	0.9189
ResNet50	2	0.9589	0.9867	0.9730	0.9728	0.9722
**ResNet50**	**3**	**0.9863**	**0.9600**	**0.9730**	**0.9732**	**0.9730**
ResNet50	4	0.9178	0.9467	0.9324	0.9322	0.9306
ResNet50	5	0.8904	0.9200	0.9054	0.9052	0.9028
InceptionV3	1	0.9589	0.8800	0.9189	0.9195	0.9211
InceptionV3	2	0.9452	0.8667	0.9054	0.9059	0.9079
**InceptionV3**	**3**	**0.9726**	**0.9067**	**0.9392**	**0.9396**	**0.9404**
InceptionV3	4	0.9178	0.9333	0.9257	0.9256	0.9241
InceptionV3	5	0.9178	0.9200	0.9189	0.9189	0.9178
**Xception**	**1**	**0.9452**	**0.9067**	**0.9257**	**0.9259**	**0.9262**
Xception	2	0.9315	0.8933	0.9122	0.9124	0.9128
Xception	3	0.9452	0.8267	0.8851	0.8859	0.8903
Xception	4	0.9178	0.9067	0.9122	0.9122	0.9116
Xception	5	0.9315	0.8133	0.8716	0.8724	0.8774

**Table 2:** Results obtained by evaluating the models with the REFUGE dataset, according to different metrics. Results corresponding to the maximum balanced accuracy (B. Accuracy) are highlighted in bold.

Network	Fold	Sensitivity	Specificity	Accuracy	B. Accuracy	F1 Score
VGG19	1	0.8000	0.8769	0.8692	0.8384	0.5501
VGG19	2	0.8167	0.8472	0.8442	0.8319	0.5117
VGG19	3	0.7333	0.9269	0.9075	0.8301	0.6132
VGG19	4	0.7833	0.8537	0.8467	0.8185	0.5054
**VGG19**	**5**	**0.8833**	**0.8898**	**0.8892**	**0.8866**	**0.6145**
ResNet50	1	0.7250	0.9620	0.9383	0.8435	0.7016
**ResNet50**	**2**	**0.8417**	**0.9009**	**0.8950**	**0.8713**	**0.6159**
ResNet50	3	0.8000	0.8981	0.8883	0.8491	0.5890
ResNet50	4	0.7833	0.9296	0.9150	0.8565	0.6483
ResNet50	5	0.8083	0.8065	0.8067	0.8074	0.4554
InceptionV3	1	0.7500	0.9435	0.9242	0.8468	0.6642
InceptionV3	2	0.8333	0.9065	0.8992	0.8699	0.6231
**InceptionV3**	**3**	**0.8500**	**0.9389**	**0.9300**	**0.8944**	**0.7083**
InceptionV3	4	0.6750	0.9843	0.9533	0.8296	0.7431
InceptionV3	5	0.7750	0.9426	0.9258	0.8588	0.6764
Xception	1	0.7500	0.9083	0.8925	0.8292	0.5825
**Xception**	**2**	**0.8083**	**0.8963**	**0.8875**	**0.8523**	**0.5897**
Xception	3	0.7000	0.9241	0.9017	0.8120	0.5874
Xception	4	0.7333	0.9148	0.8967	0.8241	0.5867
Xception	5	0.9250	0.6898	0.7133	0.8074	0.3922

**Table 3:** Results obtained by evaluating the models with the DRISHTI-GS1 dataset, according to different metrics. Results corresponding to the maximum balanced accuracy (B. Accuracy) are highlighted in bold.

Network	Fold	Sensitivity	Specificity	Accuracy	B. Accuracy	F1 Score
VGG19	1	0.8429	0.7742	0.8218	0.8085	0.8676
VGG19	2	0.8857	0.7419	0.8416	0.8138	0.8857
**VGG19**	**3**	**0.8429**	**0.8065**	**0.8317**	**0.8247**	**0.8741**
VGG19	4	0.8857	0.7419	0.8416	0.8138	0.8857
VGG19	5	0.9571	0.6452	0.8614	0.8012	0.9054
**ResNet50**	**1**	**0.9286**	**0.7742**	**0.8812**	**0.8514**	**0.9155**
ResNet50	2	0.8571	0.7419	0.8218	0.7995	0.8696
ResNet50	3	0.8143	0.7419	0.7921	0.7781	0.8444
ResNet50	4	0.9143	0.7419	0.8614	0.8281	0.9014
ResNet50	5	0.9429	0.6774	0.8614	0.8101	0.9041
InceptionV3	1	0.8429	0.8065	0.8317	0.8247	0.8741
InceptionV3	2	0.8571	0.7419	0.8218	0.7995	0.8696
InceptionV3	3	0.9000	0.7097	0.8416	0.8048	0.8873
InceptionV3	4	0.8571	0.7742	0.8317	0.8157	0.8759
**InceptionV3**	**5**	**0.8857**	**0.7742**	**0.8515**	**0.8300**	**0.8921**
Xception	1	0.8429	0.7097	0.8020	0.7763	0.8551
Xception	2	0.8714	0.6774	0.8119	0.7744	0.8652
**Xception**	**3**	**0.8286**	**0.7419**	**0.8020**	**0.7853**	**0.8529**
Xception	4	0.9143	0.6452	0.8317	0.7797	0.8828
Xception	5	0.8857	0.6774	0.8218	0.7816	0.8732

**Table 4:** Results obtained by evaluating the models with the G1020 dataset, according to different metrics. Results corresponding to the maximum balanced accuracy (B. Accuracy) are highlighted in bold.

Network	Fold	Sensitivity	Specificity	Accuracy	B. Accuracy	F1 Score
VGG19	1	0.2297	0.7459	0.5961	0.4878	0.2482
VGG19	2	0.3142	0.6630	0.5618	0.4886	0.2938
VGG19	3	0.2095	0.7845	0.6176	0.4970	0.2412
VGG19	4	0.3919	0.5939	0.5353	0.4929	0.3286
**VGG19**	**5**	**0.4628**	**0.5318**	**0.5118**	**0.4973**	**0.3549**
ResNet50	1	0.2973	0.6754	0.5657	0.4864	0.2843
**ResNet50**	**2**	**0.3547**	**0.6809**	**0.5863**	**0.5178**	**0.3323**
ResNet50	3	0.2230	0.7983	0.6314	0.5107	0.2598
ResNet50	4	0.2872	0.7169	0.5922	0.5020	0.2901
ResNet50	5	0.3919	0.6188	0.5529	0.5053	0.3372
InceptionV3	1	0.3108	0.6906	0.5804	0.5007	0.3007
InceptionV3	2	0.3986	0.5787	0.5265	0.4887	0.3282
**InceptionV3**	**3**	**0.3986**	**0.6506**	**0.5775**	**0.5246**	**0.3538**
InceptionV3	4	0.3784	0.6174	0.5480	0.4979	0.3270
InceptionV3	5	0.2905	0.7417	0.6108	0.5161	0.3023
Xception	1	0.3243	0.6865	0.5814	0.5054	0.3102
**Xception**	**2**	**0.4865**	**0.5635**	**0.5412**	**0.5250**	**0.3810**
Xception	3	0.3277	0.6823	0.5794	0.5050	0.3114
Xception	4	0.1689	0.8108	0.6245	0.4898	0.2070
Xception	5	0.3311	0.6713	0.5725	0.5012	0.3101

**Table 5:** List of the saliency methods employed in this study, along with their respective abbreviated names.

Method’s Name	Abbreviated Name
Gradient	Grad
Guided Backprop	GBack
SmoothGrad	SGrad
SmoothGrad Squared	SGrad2
VarGrad	VGrad
Integrated Gradients	IGrad
GradCAM	GCam
ScoreCAM	SCam
Occlusion	Occl

**Table 6:** Correlation coefficients for various method pairs, organized by descending median correlation values. The table provides details on each method pair, including their assigned pair number and associated minimum correlation (with its *p*-value), median correlation, and maximum correlation (with its *p*-value) values.

Pair Index #	Pair of Methods	Min. corr. val. (*p*-Value)	Med. corr. val.	Max. corr. val. (*p*-Value)
11	Grad, SGrad	0.918 (*p* < 0.001)	0.943	0.985 (*p* < 0.001)
12	Grad, SGrad2	0.623 (*p* < 0.001)	0.690	0.737 (*p* < 0.001)
19	SGrad, SGrad2	0.619 (*p* < 0.001)	0.688	0.739 (*p* < 0.001)
4	Grad, IGrad	0.250 (*p* < 0.001)	0.401	0.606 (*p* < 0.001)
14	IGrad, SGrad	0.265 (*p* < 0.001)	0.400	0.616 (*p* < 0.001)
13	Grad, VGrad	0.177 (*p* < 0.001)	0.373	0.460 (*p* < 0.001)
20	SGrad, VGrad	0.188 (*p* < 0.001)	0.365	0.461 (*p* < 0.001)
21	SGrad2, VGrad	0.213 (*p* < 0.001)	0.363	0.468 (*p* < 0.001)
15	IGrad, SGrad2	0.190 (*p* > 0.001)	0.353	0.505 (*p* < 0.001)

**Table 7:** Average results of the global relevance of the sectors, combining the results of all architectures and attribution methods. Averages are shown for both classes together and individually, and also for each dataset with which we evaluated the models (our dataset, REFUGE, DRISHTI-GS1, and G1020). Those sectors with a probability greater than or equal to 10% are highlighted in bold.

Class	Test Set	B	N	NI	TI	T	TS	NS
Both	Our dataset	**16.53%**	**19.03%**	8.35%	**13.37%**	**22.76%**	**11.20%**	8.76%
	REFUGE	**21.42%**	**21.77%**	8.78%	**10.39%**	**20.30%**	7.51%	9.82%
	DRISHTI-GS1	**15.92%**	**22.73%**	7.76%	**12.28%**	**24.93%**	**10.30%**	6.07%
	G1020	**25.80%**	**18.41%**	5.47%	8.69%	**23.63%**	9.03%	8.96%
Healthy	Our dataset	**16.46%**	**22.20%**	7.33%	9.52%	**22.41%**	**12.16%**	9.92%
	REFUGE	**18.97%**	**25.80%**	9.06%	6.43%	**18.67%**	7.96%	**13.13%**
	DRISHTI-GS1	**17.30%**	**23.38%**	8.52%	**11.63%**	**23.43%**	**10.15%**	5.59%
	G1020	**23.44%**	**19.95%**	5.32%	6.46%	**22.12%**	**10.76%**	**11.96%**
Glaucoma	Our dataset	**16.59%**	**15.86%**	9.37%	**17.22%**	**23.12%**	**10.25%**	7.60%
	REFUGE	**23.87%**	**17.75%**	8.51%	**14.36%**	**21.94%**	7.06%	6.52%
	DRISHTI-GS1	**14.55%**	**22.08%**	7.00%	**12.93%**	**26.43%**	**10.45%**	6.56%
	G1020	**28.16%**	**16.88%**	5.62%	**10.92%**	**25.14%**	7.31%	5.97%

## Data Availability

Restrictions apply to the availability of our dataset. These data are not publicly available due to legal reasons.

## Appendix A

Figure A1, Figure A2, Figure A3 and Figure A4 contained in this Appendix show the global relevance values by sectors for the different network architectures and attribution methods, as explained in Section 4.6. The data to obtain these diagrams were divided between the cases classified by the networks as healthy and those inferred as glaucoma.
